# Moral

**Published:** 1916-11-25

**Authors:** 


### MORAL.

### II. Factors in its Destruction.*

For religious people a most important reinforcement of moral is derived from the conviction that the God of Battles is fighting on the side of the soldier. Few wars have been begun, however grossly unjust, however wantonly unprovoked, without the king assuring his armies that God was on their side. The Hebrews had unhesitating confidence in the help of the Almighty in their wars; and with most primitive peoples it is considered that the honour of the national god is bound up with the success of his votaries, and that he is bound for his own reputation's sake to see that they are victorious over the votaries of some other god. In the war of 1870 the King of Prussia never issued a bulletin without assuring his troops that God was fighting for them, and in the present war the German Emperor has reverted to the more primitive attitude, and has assured his army that they, like the ancient Hebrews, have a god of their own, the good old German god, who is stronger than the god of other people, and makes it a point of honour to secure the victory of his worshippers.

Another important factor in moral is confidence in the general. This has been the decisive factor in many a battle and many a campaign. When troops have confidence in their leader they will endure anything he bids them endure, confident that whatever he orders must be right. Many a campaign that ought, by all the rules of war, to have been lost, has been won in consequence of the moral inspired in the troops, partly by direct communication from their general, partly by his example, but mainly by their confidence in him and by the corresponding diffidence which his presence inspires in the ranks of his opponents.

### Victory and Defeat.

When once a campaign is begun, and as it progresses, the main factor in the constitution of the moral is the result, as far as it has gone. Victories raise the moral by increasing the confidence in ultimate victory. Stubborn endurance is easy if it produces immediate fruits, or if past experience leads to the confident belief that it will produce immediate fruits. Nothing is more damaging to moral than defeat, and it is in defeat that moral is most needed. The great and crucial test of moral is repeated defeat, and few are the troops that can maintain a high moral under such a trial. Napoleon paid the English the compliment of saying that they never knew when they were beaten, which was equivalent to saying that their moral was unshaken by defeat; but if the English have this valuable quality, they have no monopoly of it. The Russian moral does not appear to have been shaken either by the disastrous retreat of the campaign of 1812 or by the still more disastrous retreat of 1915; and Soult's troops continued to fight with undiminished spirit and tenacity throughout the series of defeats that they suffered at the hands of Wellington in Spain and France. Nevertheless, perpetual defeat will at last destroy the moral of the best of troops. The Austrian moral was destroyed for some years after Austerlitz, the Prussian for some years after Jena, and after the Nile the English and French ships rarely met without confidence of victory on the one side and certainty of defeat on the other. The most splendid and conspicuous examples of moral, sustained in the face of circumstances the most adverse and disastrous, have been furnished by sieges. There are few nations that cannot boast of sieges endured with heroic and almost incredible stubbornness, in spite of continual encroachment by the besiegers, of famine, of the ravages of disease, and of sufferings endured by the combatant garrison.

If we view the circumstances of the present war for the purpose of discovering the moral of the enemy, we find much that is encouraging. The most important and the most significant circumstance is that the Government of Germany has itself no confidence in the moral of either the army or the people of Germany The German Government is, we know, stupid and inefficient in estimating the minds of other nations; but it can scarcely be so stupid or so inefficient as not to read aright the mind of Germany, and the moral of Germany it does not trust. It has no confidence that Germans will endure stubbornly, for it dares not tell them the truth. It is hoping against hope that some turn in the tide of affairs, a victory, the detachment of one of the Allies, a proposal of peace from a neutral, something or other, will obviate the necessity of revealing the truth; but day by day the inexorable necessity of revealing the truth draws nearer and nearer. If it is at length revealed by the Government, what will happen? If it leaks out and becomes known in spite of the frantic denials of the Government, what will happen? No one can say with any assurance, but indications are not wanting that in spite of the slavish submission of Germans to their Government they are as apt to demand scapegoats, even from among the members of the Government, as other and freer peoples. Moltke is gone, Tirpitz is gone, Falkenhayn is gone, a dead-set is being made against Bethmann-Hollweg. When all are gone, and the new men do no better than the old, what will happen? Who will be the next scapegoat? Will not some threatened scapegoat, in order to save his own skin, reveal the truth, and expose the whole tissue of deceit and concealment? And when the German people learn that this war in which they have suffered so frightfully, and in which they are still to suffer so frightfully, was not forced upon them, was not a defensive war, but was deliberately planned and forced upon the world by their own Government, what will happen? The truth cannot be concealed for ever; and though the German is towards his own Government nothing but a sheep, no lion is so dreadful as a *mouton enragé*.

* Article I. was published on November 18.

